# Применение радиоактивных веществ в медицине — история и перспективы развития

**DOI:** 10.14341/probl12824

**Published:** 2021-12-30

**Authors:** М. С. Шеремета, А. А. Трухин, М. О. Корчагина

**Affiliations:** Национальный медицинский исследовательский центр эндокринологии; Национальный медицинский исследовательский центр эндокринологии; Первый Московский государственный медицинский университет им. И.М. Сеченова

**Keywords:** ядерная медицина, радиоактивные изотопы йода, ионизирующее излучение, радиойодтерапия, щитовидная железа, тиреотоксикоз, рак щитовидной железы

## Abstract

Ядерная медицина (ЯМ) – медицинская специальность, использующая радионуклиды (радиоактивные “трейсеры”) и ионизирующее излучение с диагностико-терапевтической (тераностической) целью. ЯМ возникла и развивается на стыке физики, химии и клинической медицины. Излучение, испускаемое радиоактивными изотопами, может состоять из гамма-лучей, бета- и альфа-частиц или их комбинации. Выбор радиоизотопа для медицинских целей осуществляется в соответствии со следующими требованиями: низкая радиотоксичность, подходящий тип радиоактивного излучения, энергия и период полураспада (от нескольких минут до нескольких часов и дней), а также удобное для регистрации гамма-излучение. Радионуклид входит в состав радиофармацевтического лекарственного препарата (РФЛП) и выполняет роль его маркера. РФЛП накапливается в анатомических структурах, становится переносчиком координированной информации от пациента к гамма-камере или другому медицинскому прибору и отражает динамику процессов, протекающих в исследуемом органе. В 2021 году ЯМ отмечает 80-летний юбилей. Траектория развития ЯМ объединяет современные методы радиотераностики, включая прикладные геномные и постгеномные технологии.

## Abstract (en)

Nuclear medicine (NM) is a medical specialty that uses radionuclides (radioactive tracers) and ionising radiation for diagnostic and therapeutic (theranostic) purposes. Nuclear medicine arose and developed at the intersection of physics, chemistry and clinical medicine. The radiation emitted by radioisotopes can consist of gamma-, beta- and alpha emission, or it’s combination. Radioisotope of choice for medical purposes should have futher requirements: low radiotoxicity, suitable type of radiation, energy and half-life (several minutes to several hours and days), and also convenient detection of gamma ray radiation. The radionuclide is part of radiopharmaceutical (RP) and acts as its indicator. RP accumulates in morphological structures, becomes a carrier of coordinated information from patient to gamma camera or other equipment and reflects the dynamics of processes occurring in the examined organ. In 2021 NM celebrates its 80th anniversary. The trajectory of NM combines modern methods of radiotheranostics and applied genomic and post-genomic technologies.

## ВВЕДЕНИЕ

Фундаментальные исследования, проведенные в ядерной физике в 1920–1940-х гг., положили начало радиационной и ЯМ. Период зарождения ЯМ располагается между открытием искусственной радиоактивности в 1934 г. и производством радионуклидов Oak Ridge National Laboratory, США, в 1940 г. для их использования в медицинских целях. Потребовалось немало усилий, прежде чем применение радиоактивности стало безопасным и эффективным.

Один из первых искусственно полученных радиоизотопов, используемых в медицине вот уже 80 лет, — изотоп 131I. Его выделяют в форме йодида натрия (NaI) из продуктов распада урана или нейтронной бомбардировки теллура-130 в ядерном реакторе. Изотоп 131I излучает β- и γ-лучи, период полураспада — 8,02 дня (рис. 1) [1].

**Figure:**
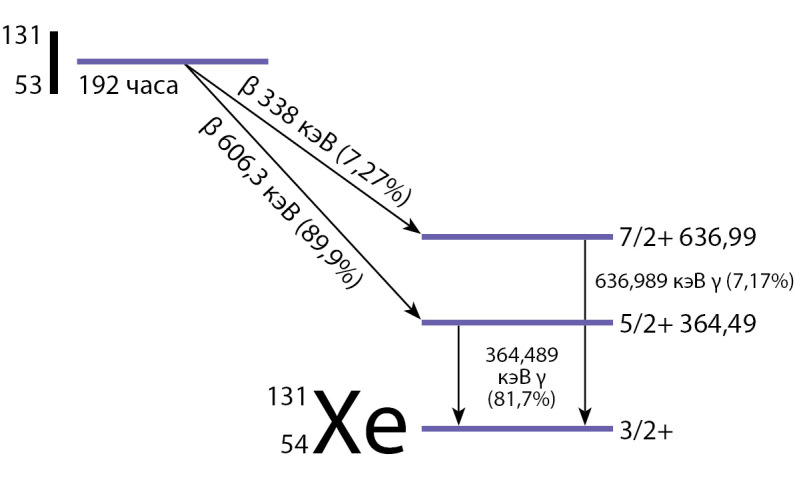
Рисунок 1. Схема распада 131I. Изотоп 131I распадается в стабильный 131Xe (131-Ксенон) в два этапа, при этом γ-распад следует сразу после β-распада. Основная энергия γ-излучения равна 364 кэВ (89,9%), β-излучения — 606 кэВ (81,7%). С меньшим выходом наблюдается γ-излучение с энергией 637 кэВ (7,2%), β-излучение с энергией 338 кэВ (7,3%).

Новая медицинская парадигма, тераностика, использует один многофункциональный агент для терапии и диагностики, достигая максимального персонализированного терапевтического ответа, а также улучшая долгосрочный прогноз и снижая дозозависимую токсичность. Термин «тераностика» образован путем слияния слов «терапия» и «диагностика». Он был введен в 2002 г. американским консультантом John Funkhouser, главным исполнительным директором PharmaNetics [2]. Один из первых тераностических агентов, радиоактивный йод, использован при заболеваниях щитовидной железы (ЩЖ) в 1940-х гг., а в 1950 г. американским врачом Benedict Cassen проведена первая визуализация щитовидной железы (ЩЖ) с использованием прямолинейного сканера после введения в организм радиоактивного йода [3].

## ЙОД: РОЛЬ, МЕТАБОЛИЗМ

Основную роль в метаболизме йода в организме играет ЩЖ. Йод — важный микроэлемент, необходимый для синтеза гормонов ЩЖ, тироксина (Т4) и трийодтиронина (Т3). Йод составляет 65 и 59% массы Т4 и Т3 соответственно [4]. Выработка и секреция гормонов ЩЖ регулируются тиреотропным гормоном (ТТГ) передней доли гипофиза.

В организме здорового взрослого человека содержится 15–20 мг йода, из них 70–80% находится в ЩЖ. В основном йод поступает в организм с пищей, при этом наиболее богатые йодом продукты — это йодированная соль, молочные продукты, некоторые виды хлеба, морские водоросли и морепродукты. При хроническом дефиците йода его содержание в ЩЖ может снизиться до менее чем 20 мкг. В районах с достаточным потреблением йода ЩЖ взрослого человека захватывает около 60 мкг йода в день, чтобы сбалансировать потери и поддерживать синтез Т4 и Т3 [4].

После всасывания йод выводится из основного кровотока главным образом ЩЖ и почками. Поглощение йода ЩЖ обеспечивается Na+/I–-симпортером, описанным S. Kaminsky и соавт. в 1993 г. [5]. Na+/I–-симпортер располагается на базолатеральной мембране клеток ЩЖ. Перенос йодида из циркулирующей крови внутрь тиреоцита происходит по градиенту концентрации, который образуется Na+/K+-АТФазой и примерно в 30–50 раз превышает концентрацию в плазме, обеспечивая поступление достаточного количества йода для синтеза тиреоидных гормонов [4][6].

ТТГ регулирует транспорт йода в ЩЖ, стимулируя транскрипцию Na+/I–-симпортера и способствуя его правильному встраиванию в плазматическую мембрану. При диете с низким содержанием йода ТТГ стимулирует экспрессию Na+/I–-симпортера и отвечает за изменение его субклеточной локализации.

Существует механизм, обеспечивающий нормальное функционирование ЩЖ при избытке йода. Когда в организм человека с нормальной функцией ЩЖ поступает большое количество йода, происходит транзиторное снижение синтеза тиреоидных гормонов в течение примерно 48 ч. Избыток йода блокирует его органификацию и образование Т3, Т4 [7]. Этот процесс, описанный в 1948 г., называется эффектом Вольфа–Чайкова [8]. Предполагается, что эффект Вольфа–Чайкова связан с негативной посттрансляционной регуляцией Na+/I–-симпортера йодидами [9]. В ответ на введение больших количеств йода уменьшается высвобождение тиреоидных гормонов из ЩЖ с компенсаторным увеличением уровня ТТГ. Со временем синтез тиреоидных гормонов восстанавливается. Феномен «ускользания» от эффекта Вольфа–Чайкова является адаптационным процессом, он не зависит от ТТГ и связан со снижением содержания йода внутри ЩЖ. Наиболее вероятный механизм развития этого явления — снижение активности Na+/I–-симпортера, приводящее к уменьшению поступления микроэлемента в тиреоциты. Когда уровень йода в ЩЖ становится ниже значений, поддерживающих эффект Вольфа–Чайкова, процесс органификации восстанавливается, а вместе с ним нормализуется функция ЩЖ [7].

Считалось, что йодид пересекает апикальную мембрану под действием электрохимического градиента, однако исследования показали, что ТТГ стимулирует перенос йодида через апикальную мембрану специфическим транспортером — пендрином. Пендрин принадлежит к семейству SLC26A и кодируется геном SLC26A4. Он присутствует в апикальной мембране фолликулярных клеток [4].

В просвете фолликулов йодид окисляется и включается в тиреоглобулин (ТГ) тиреоидной пероксидазой — ферментом, локализованным в микросомальной фракции фолликулярных клеток ЩЖ. Этот процесс называется органификацией и приводит к образованию 3-монойодтирозина и 3,5-дийодтирозина. После тирозольные остатки сближаются друг с другом и конденсируются, образуя йодтиронины — тиреоидные гормоны. Йодированный ТГ хранится в просвете фолликула в густой жидкости, которая называется коллоидом и выводится посредством эндоцитоза или микропиноцитоза с последующим протеолитическим расщеплением в лизосомах и высвобождением Т3 и Т4 в кровоток [10].

## ТЕРАПИЯ РАДИОАКТИВНЫМ ЙОДОМ — ПРИМЕР УСПЕШНОЙ ИСТОРИИ ЯДЕРНОЙ МЕДИЦИНЫ

Современное представление о применении радиоактивности основано на знаниях физических и радиобиологических свойств изотопов, применении концепции тераностики с возможностью достижения максимально эффективного и безопасного лечения. Мультидисциплинарная работа команды врачей, физиков и радиохимиков началась более 80 лет назад. Потребовалось много усилий, прежде чем радиойодтерапия (РЙТ) стала стандартным радикальным методом лечения доброкачественных и злокачественных заболеваний ЩЖ.

Первые открытия

Эру применения ионизирующих излучений отсчитывают с 1895 г., когда немецкий физик Wilhelm Conrad Röntgen в экспериментах с трубкой Крукса открыл рентгеновское излучение. Шесть лет спустя — в 1901 г. — за это открытие Röntgen получит первую в истории Нобелевскую премия по физике.

В 1896 г. Jules Henri Poincare, французский ученый широкого профиля, на заседании Парижской Академии высказал предположение, что рентгеновское излучение связано с флуоресценцией и, возможно, всегда возникает в люминесцирующих веществах, поэтому катодной трубки для получения рентгеновских лучей не требуется.

В этом же году данную гипотезу проверил французский физик Antoine Henri Becquerel [1]. Он использовал фотографическое действие через черную бумагу активированных солнечным светом кристаллов соли урана. На первом этапе эксперимент подтвердил гипотезу H. Poincare, но вскоре A. Becquerel обнаружил, что урановая соль без воздействия солнечного света способна испускать излучение, проходящее через черную бумагу.

Незадолго до этого открытия, в 1895 г., признана роль йода как предшественника гормонов ЩЖ. Немецкий химик Eugen Baumann пролил концентрированную азотную кислоту на образец ЩЖ и увидел пурпурные испарения, поднимающиеся над ней. Так стало известно, что стабильный йод1 присутствует в тканях ЩЖ [4].

Прежде чем радиоактивные изотопы (РИ) йода заняли свое почетное место в истории ЯМ, были изучены свойства таких радиоэлементов, как уран, торий, радий, полоний, актиний. Большой вклад в изучение радиоактивности внесли польская и французская ученая-экспериментатор Marie Curie и ее муж — французский физик Pierre Curie. В 1903 г. Шведская королевская академия наук присудила Нобелевскую премию по физике A. Becquerel и супругам Curie «за выдающиеся заслуги в совместных исследованиях явлений радиации» [14].

В 1923 г., в то время как будущий врач и отец тераностики Saul Hertz учился в Гарвардской медицинской школе, венгерский химик György de Hevesy создал принципы использования радиоактивных веществ для изучения биологических процессов. Позднее, в 1934 г., итальянский физик Enrico Fermi путем облучения альфа-частицами алюминиевой фольги синтезировал 22 новых радиоактивных изотопа, среди которых были изотопы йода [13][15].

В 1931 г. Saul Hertz был принят на работу в Клинику щитовидной железы и Лабораторию метаболизма Массачусетской больницы общего профиля (MGH). Ранее Saul Hertz являлся волонтером, а впоследствии стал заведующим Клиникой щитовидной железы [16].

Karl Compton: «Что физика может сделать для биологии и медицины»

Saul Hertz знал, что йод легко поглощается ЩЖ и некоторые химические элементы можно сделать радиоактивными, но о работах Enrico Fermi ему было неизвестно. В 1936 г. Karl Compton, американский физик и президент Массачусетского технологического института (MIT), выступил на обеде в Вандербиль холле с докладом «Что физика может сделать для биологии и медицины», что послужило исторической точкой в зарождении исследования РИ йода [17][18]. Karl Compton был старшим братом Arthur Compton, физика, который в 1923 г. открыл явление рассеяния электромагнитного излучения на свободных электронах (известное как «эффект Комптона»). Возможно, в своем докладе Karl Compton затронул открытие явления искусственной радиоактивности, сделанное французскими физиками Frederic и Irene Joliot-Curie в 1934 г. [17].

Группа клиницистов и ученых, присутствовавших на обеде, вдохновилась выступлением Karl Compton и захотела больше узнать о радиоактивном йоде. Saul Hertz поинтересовался: «Можно ли искусственным путем сделать йод радиоактивным?» Несколько лет он изучал роль йода в физиологии ЩЖ в MGH в поисках нехирургического решения проблемы тиреотоксикоза. По другим рассказам, главный врач MGH, профессор James Howard Means, в конце доклада поинтересовался фактической биодоступностью радиоактивных изотопов йода. Он также хотел понять, может ли MIT производить РИ йода искусственным путем [17].

Karl Compton не имел однозначного ответа, возможно, потому, что его лекция об искусственных радиоизотопах была инициирована в последнюю минуту Robley Evans, американским физиком из MIT [19]. Он записал вопрос Hertz и ответил ему в письме спустя месяц, 15.12.1936: «К моему сожалению, я только сейчас наткнулся на заметку, сделанную по вашему вопросу о радиоактивном йоде. Йод можно сделать радиоактивным искусственно. Он имеет период полураспада в 25 минут и испускает γ- и αβ-лучи (электроны) с максимальной энергией 2,1 миллиона вольт» [20]. Период полураспада в 25 минут соответствует изотопу йода с атомной массой 128, т.е. 128I.

Hertz ответил на письмо через 8 дней — 23.12.1936. Выдвигая свою гипотезу, он писал: «...надеюсь, что йод, который, как вы указали, можно сделать радиоактивным, будет полезным методом терапии в случаях гиперфункции щитовидной железы». Предполагается, что эта гипотеза построена на более ранних работах Howard Means 1922 г. относительно возможного лечения заболеваний ЩЖ с помощью рентгеновского излучения. Обмен письмами между Compton и Hertz — бесспорное доказательство того, что идея применения РЙТ возникла именно у эндокринолога Saul Hertz.

Перспективное сотрудничество

В 1937 г. Hertz стал сотрудничать с молодым физиком и преподавателем Arthur Roberts, с которым в ходе последующих экспериментов изучал возможность применения радиоактивного йода (РЙ) для диагностики и лечения заболеваний ЩЖ.

Основываясь на работе Fermi, будущего создателя ядерного реактора, группа построила самодельный источник нейтронов, который содержал бериллий и переработанный радий (Ra-Be). Спиртовой раствор йода (127I) облучался в течение 1 или 2 ч путем погружения его в источник нейтронов с целью создать преципитат радиоактивного иодида серебра (Ag128I) [17].

Первые эксперименты на животных моделях включали изучение 48 кроликов2, которым вводили Ag128I.

Все животные выжили. Поглощение радиоактивного йода (RAIU) изучили при различных состояниях функции ЩЖ. Затем радиоактивность тканей животных измеряли с помощью счетчика Гейгера–Мюллера. Roberts использовал счетчик Гейгера-Мюллера для определения количества РИ йода, присутствующего в биологических образцах. Результаты показали, что RAIU ЩЖ происходит быстро и зависит от степени гиперплазии3.

Hertz и Roberts смогли провести исследования фармакокинетики йода, а в мае 1938 г. ими опубликована первая статья под названием «Радиоактивный йод как индикатор в изучении физиологии щитовидной железы» [21]. Исследования показали, что РЙ может использоваться для оценки функции ЩЖ.

Циклотрон и изотопы радиоактивного йода

За несколько лет до этих экспериментов американский физик Ernest Orlando Lawrence и его команда из Беркли разработали циклотрон для исследований в области ядерной физики. После открытия Joliot–Curie стало очевидно, что циклотрон можно применять для создания искусственной радиоактивности [22].

В 1938 г. американские ученые Glenn Seaborg и John Livingood получили 131I путем облучения источников теллура [23]. Этот прорыв произошел после разговора Seaborg с Joseph Hamilton, профессором медицинской физики, экспериментальной медицины и радиологии. Hamilton объяснил, что короткий период полураспада 128I ограничивает клиническое применение данного изотопа. Преимущество же 131I в том, что он излучает как γ-, так и β-лучи: первые позволяют получать изображения для диагностики и дозиметрии, вторые применимы для таргетной радионуклидной терапии. Данная особенность 131I делает его первым естественным тераностическим радионуклидом в медицине.

Параллельно с Hertz и Roberts ученые Joseph Hamilton и Mayo Soley проводили свои эксперименты с использованием РЙ и других радиоактивных элементов [24]. К июню 1939 г. они исследовали RAIU ЩЖ человека и предположили, что можно получить динамическую картину метаболизма йода, оценив уровни поглощения и выделения йода ЩЖ4. Команда исследователей сообщила об отсутствии RAIU у больного со злокачественным образованием ЩЖ. В 1940 г. было подтверждено, что ЩЖ человека захватывает радиоактивный йод.

Первая оценка опытов Hertz и Roberts привела к лучшему пониманию метаболизма йода при болезни Грейвса и была представлена на заседании Американского общества клинических исследований (ASCI) в Атлантик-Сити в мае 1942 г. Joseph Hamilton также присутствовал на встрече и сообщил о своем опыте лечения РЙ (130I) трех пациентов с тиреотоксикозом [26].

Первые результаты терапии радиоактивным йодом

Пациентка ED направляется в клинику доктора Hertz с тиреотоксикозом без офтальмопатии и с уровнем базального метаболизма5 (BMR) +30.

31 марта 1941 г. ей назначают активность 77,7 МБк смеси 130I/131I, а потом дополнительные 48,1 МБк. ED становится первой пациенткой с заболеванием ЩЖ, которую лечат РИ йода. По совету James H. Means, ED получает стабильный йод вслед за дозой РЙ. После терапии BMR пациентки снижается до -7. Об этом клиническом случае было сообщено в мае 1941 г. на ежегодном собрании ASCI [18].

В течение последующих 2 лет (1941–1943 гг.) Hertz и Roberts пролечили 29 пациентов с тиреотоксикозом и после терапии вводили всем нерадиоактивный йод. Сообщалось, что 20 пациентов считались вылеченными, 9 без положительного ответа на лечение были направлены на хирургическое лечение, а при патологоанатомическом исследовании образцов ЩЖ у 6 из них выявлена инволюция. Однако эти результаты будут сообщены только через несколько лет — в 1946 г. Причина тому — война.

В 1943 г., в разгар Второй мировой войны, доктор Saul Hertz решил добровольно принять участие в военной службе и поступил на службу в Военно-морской флот США. Пока Hertz отсутствовал, Earl Chapman, врач частной практики из MGH, работал с Evans над лечением пациентов с заболеваниями ЩЖ. На этот раз за введением радиоактивного йода не последовало введения дозы стабильного йода.

Сенсация

Hertz вернулся домой в 1945 г. Он не смог продолжить свои исследования в MGH из-за разногласий, возникших с Chapman, и присоединился к докторам больницы Beth Israel.

Chapman первым отправил публикацию в Журнал Американской Медицинской ассоциации (JAMA). Но она была слишком длинной, и ее вернули на доработку. В ноябре 1945-го главный редактор JAMA, американский врач Morris Fishbein, сообщил Hertz, что Клиника щитовидной железы представила рукопись по лечению РИ йода без упоминания Hertz и Roberts, что побудило Hertz быстрее закончить свою работу по РЙТ.

Необычное обстоятельство — две рукописи на одну тему из одного учреждения без перекрывающегося авторства были опубликованы в одном медицинском журнале 11 мая 1946 г. Это подтверждало, что РЙТ — перспективный метод лечения тиреотоксикоза [27][28].

Изотоп 131I появился в свободном доступе с 1946 г. в результате реализации манхэттенского проекта в Ок-Ридж в Теннесси [19].

Возможность исследовать метаболизм и функцию ЩЖ с помощью РИ йода использовалась для лечения доброкачественных и злокачественных заболеваний ЩЖ. В 1946 г. Hertz был принят в ASCI в качестве Young Turk за революционную работу по разработке первой тераностической методики в медицине: использование РИ йода при заболеваниях ЩЖ [19].

Применение РЙТ при злокачественных образованиях ЩЖ

Первыми исследованиями применения РИ йода при карциноме ЩЖ стали клинические работы под руководством врача-ученого Hamilton. В исследовании 1942 г. команда Hamilton ввела РИ йода 2 пациентам с тиреотоксикозом, которым впоследствии была сделана ТЭ. Патологоанатомическое исследование образца ЩЖ показало диффузное накопление РЙ в злокачественных очагах железы [29].

В том же году доктора Reid и Albert S. Keston открыли 125I, а позже Keston и его коллеги из Колумбийского университета сообщили о поглощении РЙ (RAIU) метастазами карциномы ЩЖ [30]6. Результаты имели большое значение в понимании потенциальной роли абляции остаточной ткани ЩЖ с помощью РЙ.

Клиническое применение РЙ для диагностики и лечения дифференцированного рака щитовидной железы (РЩЖ), проведенное в 1942 г., принадлежит американскому эндокринологу Samuel M. Seidlin, который отметил, что поглощение радиоактивного йода метастазами можно индуцировать инъекцией рекомбинантного человеческого тиреотропина или тиреоидэктомией (ТЭ) [32]. 7 декабря 1946 г. JAMA опубликовала обширный отчет Seidlin о первом успешном лечении метастатической карциномы ЩЖ с использованием РЙ [33].

В 1946 г. Hertz заявил, что его исследования будут сосредоточены на «раке щитовидной железы, который является ключом к более серьезной проблеме — онкологии в целом» [19].

У одной из первых пациенток, поступивших в Королевский онкологический центр (Лондон) в 1949 г., был неоперабельный дифференцированный РЩЖ и метастазы, требующие трахеостомии. Первая диагностическая активность в 2,59 МБк 131I получена. На фоне хорошего поглощения РЙ удалось назначить терапевтические активности. Спустя несколько сеансов (1850, 3256, 3700, 8510 МБк) РЙ, выполненных в течение 7 мес, отмечалось улучшение состояния пациентки7.

Клинический случай

Пациент BB, мужчина 51 года со злокачественным образованием ЩЖ. Из анамнеза известно: в возрасте 30 лет (командой врачей Seidlin; Montefiore Hospital, Нью-Йорк, США) пациенту выполнена тотальная ТЭ большого зоба, сопровождающегося компрессионным синдромом. Микроскопическое исследование образца ЩЖ не выявило нормальных структур, а патологоанатомический диагноз звучал как злокачественная аденома. После операции полного удаления ткани ЩЖ пациент находился в ремиссии на протяжении 15 лет, пока у него не появились классические симптомы тиреотоксикоза. Мужчина сильно похудел, жаловался на учащенное сердцебиение и тревожность. Симптомы нарастали, а в ноябре 1939 г. мужчина был направлен на хирургическое лечение из-за пульсирующей опухоли на уровне TXII. Уровень его BMR достиг +40. Выполнена ламинэктомия TXII и LI для эксцизионной биопсии, в результате которой выявлена метастатическая карцинома ЩЖ.

Послеоперационный период осложнился тиреотоксическим кризом. Состояние пациента ухудшалось. Команда Seidlin использовала ежедневные дозы 1–6 мл р-ра Люголя в течение почти 10 мес, блокируя органификацию йода, подавляя присоединение молекулярного йода к ТГ и образование тиреоидных гормонов Т3 и Т4. Сначала BB показал симптоматическое улучшение, но к январю 1943 г. его состояние ухудшилось. Seidlin незамедлительно проконсультировался с Hertz, чтобы обсудить возможность использования РЙ для лечения метастазов. Организовать поставку РЙ оказалось крайне трудно. Evans запросил огромную сумму — 1800 долларов за час — и поинтересовался, сколько миллиКюри нужно доктору. «Пришлите мне за час», — ответил Seidlin. Позднее Seidlin признал, что ни у него, ни у пациента не было 1800 долларов, кроме того, он не понял, что Evans имел в виду под «миллиКюри» [19].

Первая терапевтическая активность РЙ, введенная 11 мая 1943 г., содержала 629 МБк 130I. В общей сложности пациент получил 16 терапевтических активностей 130I и 131I. Суммарная активность составила 9945 МБк. Доктора отслеживали выделение РЙ мочой. Из-за непомерной стоимости радионуклида, генерируемого циклотроном, они извлекли и переработали РЙ из мочи пациента для повторного использования. Команда Seidlin наблюдала клиническое улучшение больного в самом начале и при каждом последующем применении РЙТ. Пациент набрал вес, боль в костях почти прошла, беспокоящее сердцебиение прекратилось, а BMR упал до –27. Доктора были поражены результатами лечения. Клинический случай опубликован в октябрьском номере журнала LIFE в 1949 г. В статье несколько приукрасили результаты, сообщая о том, что мужчина полностью выздоровел. На самом деле спустя несколько лет пациент умер от установленной при вскрытии анапластической карциномы [19].

В 1949 г. доктор Hertz создал первое отделение ЯМ, расширив исследования с использованием радионуклидов в области онкологии [17]. В январе 1950 г. опубликована исчерпывающая глава, в которой сообщалось о применении РЙТ при карциноме ЩЖ. «В целом результаты использования РЙТ при злокачественных образованиях щитовидной железы хотя и многообещающие, но не свидетельствуют о большом проценте излечений за короткое время, в течение которого используется метод», — писал Hertz [35]. Он понимал, что потребуется время для лучшего понимания возможностей РЙТ у пациентов с раком ЩЖ. В июле 1950 г. Hertz умер в результате внезапного сердечного приступа.

В 1951 г. 131I стал первым радиофармацевтическим препаратом, получившим одобрение Food and Drug Administration (FDA), который использовался для лечения заболеваний ЩЖ. Показания для применения РЙТ при злокачественных образованиях ЩЖ сформулированы в 1957 г. американским врачом William Henry Beierwaltes, пионером в использовании ЯМ: «Желательно провести РЙТ для того, чтобы закончить работу, которую начал хирург». Определена цель применения РЙТ при РЩЖ — полная абляция ткани ЩЖ [19].

## РЙТ — МЕХАНИЗМ ДЕЙСТВИЯ 131I И ЛЕЧЕНИЕ ЗАБОЛЕВАНИЙ ЩЖ

Поглощение и метаболизм радиоактивного йода 131I в ткани ЩЖ идентичны стабильному йоду. При пероральном приеме 131I (капсула или раствор) в виде йодид-иона всасывается из желудочно-кишечного тракта. Na+/I–-симпортер транспортирует йодид клетками ЩЖ через базолатеральную мембрану фолликулярных клеток. Некоторые экстратиреоидные ткани (слизистая оболочка желудка, слюнные, слезные и лактирующие молочные железы), также содержащие Na+/I–-симпортер, опосредованно поглощают 131I через базолатеральную мембрану фолликулярных клеток.

Экспрессия белка Na+/I–-симпортера усилена при болезни Грейвса, что подтверждается повышенным поглощением РИ йода. При многоузловом токсическом зобе экспрессия белка Na+/I–-симпортера выше, чем в норме, и ниже, чем при болезни Грейвса. При раке ЩЖ экспрессия NIS (SLC5A5), как правило, снижена [6][9].

Попадая в фолликулярные клетки ЩЖ, 131I включается в состав фенольного кольца тирозильных остатков тиреоглобулина. Когда β-частицы 131I высвобождаются внутри клеток ЩЖ, они проходят в среднем 0,8 мм, прежде чем их энергия полностью поглощается. β-частицы, испускаемые 131I, разрушают функционирующую ткань ЩЖ. Радиобиологическое воздействие 131I на ткани бывает прямым (влияние радиации на ДНК — разрыв молекулярных связей) или косвенным. При косвенном воздействии образуются свободные радикалы, которые вступают в реакцию с макромолекулами в среде микроокружения клеток. Свободные радикалы и оксиданты взаимодействуют с молекулами ДНК, вызывая большое количество разнообразных нарушений ее структуры, обеспечивая локальную деструкцию тиреоцитов. Воздействие ионизирующего излучения приводит к генетическим повреждениям, мутациям или гибели клеток. Таким образом, 131I вызывает обширное местное повреждение и некроз тканей ЩЖ. Основной эффект РЙТ — радиационный тиреоидит. Тяжесть этого эффекта прямо пропорциональна полученной дозе [36]. Целью терапии является подавление функционального состояния клеток и нарушение способности к пролиферации [37]. Эффективная абляция тиреоидной ткани происходит через 8–16 нед с отсутствием возможности выработки тиреоидных гормонов [9, 38].

## РОЛЬ NA+/I–-СИМПОРТЕРА В ЛЕЧЕНИИ ЗАБОЛЕВАНИЙ ЩЖ С ИСПОЛЬЗОВАНИЕМ 131I. ЙОДРЕЗИСТЕНТНОСТЬ

РЙТ основана на способности клеток ЩЖ захватывать и накапливать 131I. Свойство накопления 131I может отсутствовать примерно в 10% случаев высокодифференцированного РЩЖ. В процессе дифференцировки клетки ЩЖ теряют способность накапливать 131I, что делает проведение РЙТ неэффективным лечением [39]. Есть данные, что 18–25% дифференцированных карцином ЩЖ исходно лишены способности к захвату 131I, а 35–50% пациентов перестают реагировать на лечение 131I во время РЙТ [40].

Дисбаланс работы Na+/I–-симпортера, а именно снижение его экспрессии, дефекты белка Na+/I–-симпортера и нарушение его встраивания в плазматическую мембрану — ключевые факторы развития резистентности к РЙТ, приводящие к неэффективности лечения. Мутации в гене NIS и в других генах, таких как RAS (KRAS, HRAS, NRAS), TERTp и BRAF, могут способствовать более низкой экспрессии мРНК и белка Na+/I–-симпортера, а также нарушениям его ориентации на мембране [41]. Описанные генетические альтерации ведут к снижению поглощения 131I и высокому риску рецидива РЩЖ после РЙТ. Как пример, при наличии мутации в гене BRAF представляется возможным использование таргетных препаратов, ингибиторов RAF-киназ (дабрафениб) и MEK (траметиниб), регулирующих MAP-киназный путь. К другим молекулярным маркерам, участвующим в канцерогенезе, относятся хромосомные перестройки RET/PTC, PAX8/PPARG и мутации в генах TP53, PIK3CA и AKT1.

Есть как минимум два ключевых агента, контролирующих транспорт и количество Na+/I–-симпортера, — фактор рибозилирования АДФ 4 (ARF4) и валозин-содержащий белок (VCP). ARF4 усиливает везикулярную транспортировку Na+/I–-симпортера от аппарата Гольджи к плазматической мембране, а VCP, находящийся на эндоплазматическом ретикулуме и отвечающий за деградацию, регулирует протеолиз Na+/I–-симпортера. Известно, что экспрессия VCP повышена при агрессивных формах течения РЩЖ и у пациентов с неблагоприятным прогнозом после РЙТ, а продолжительность жизни пациентов радиойодрезистентным РЩЖ, особенно при метастатическом поражении, составляет 3–5 лет [41][42].

В экспериментах Fletcher и соавт. абляция ARF4 в NIS+ клетках TPC-1, а также в первичных тиреоцитах человека привела к снижению поглощения 125I от ∼60% до 80%, а сверхэкспрессия ARF4 — к значительному увеличению поглощения 125I. Индукция VCP в этих клетках путем транзиторной трансфекции способствовала подавлению поглощения 125I. Подтверждено, что и ARF4, и VCP регулируют ориентацию Na+/I–-симпортера. Экзогенная экспрессия ARF4 в NIS+-клетках демонстрирует удвоение количества белка Na+/I–-симпортера на плазматической мембране, при этом сверхэкспрессия VCP приводит к его значительному снижению.

Специфических агонистов ARF4 в настоящее время не существует, но можно повлиять на Na+/I–-симпортер путем ингибирования VCP. Два ингибитора VCP, одобренных FDA, а именно клотримазол и эбастин, подавляют VCP-опосредованное снижение активности Na+/I–-симпортера (специфическое ингибирование и аллостерическая регуляция) и способствуют увеличению количества Na+/I–-симпортера на поверхности клеток, а также повышают поглощение РЙ в моделях ЩЖ мыши и человека in vitro [41]. Таким образом, возможно повлиять на успех РЙТ у пациентов с радиойодрезистентным РЩЖ с помощью данной группы препаратов.

К более известным таргетным препаратам, одобренным FDA и применяемым при радиойодрезистентном РЩЖ, относятся мультикиназные ингибиторы сорафениб и ленватиниб [43]. Они блокируют ангиогенез в опухолевой ткани и ингибируют киназы, задействованные в онкогенных механизмах, тем самым замедляя рост опухоли.

## ОБСУЖДЕНИЕ

Многолетний опыт применения радиоактивного йода, современные знания радиобиологии, тераностики и молекулярной генетики составляют основу эффективного и безопасного применения радиоактивности. Изучение фармакокинетики, сопоставление клинических предикторов течения болезни и выбранной модели расчета индивидуальной терапевтической активности позволяют персонализированно использовать радиоизотопы. Оценка фармакодинамики как элемента радиобиологии важна при сопоставлении поглощенной дозы функционирующей ткани ЩЖ с эффектом лечения. Фармакобезопасность радионуклидной терапии основана на совершенствовании методов профилактики вторичных осложнений, снижении лучевой нагрузки и рисков отдаленных эффектов. Технологическое дозиметрическое обеспечение в перспективе позволит более детально описывать поведение йода не только в ткани ЩЖ, но и в организме в целом с учетом накопления в тропных органах. Спектрометрическое оборудование способно определить низкое содержание 131I в образце крови, что позволяет проводить биодозиметрию крови с целью определения максимально допустимой терапевтической активности при проведении радиойодтерапии дифференцированного РЩЖ. Совершенствование оборудования ЯМ, способов ранней диагностики заболеваний ЩЖ и профилактики детерминированных и отсроченных клинических проявлений РЙТ — ключ к дальнейшему развитию технологии персонализированной медицины.

Дозиметрическое планирование радиойодтерапии

В совместной работе американских ученых — медицинских физиков E. Quimby и L. Marinelli — представлен опыт оптимизации лечения с использованием радиоактивных веществ.

E. Quimby в 1951 г. представила сферические модели органов, необходимых для расчета поглощенных доз излучения. Наблюдения позволили сделать вывод, что захват исследуемой ткани в фиксированное время после введения сильно зависит от особенностей фармакокинетики человека.

L. Marinelli совместно с E. Quimby акцентировали внимание на важных для расчета терапевтической активности 131I характеристиках фармакокинетики и физико-математического моделирования распространения элементарных частиц. Они предложили максимально простую реализацию комбинации параметров в виде формулы, учитывающей объем ЩЖ, индекс захвата 131-йода, эффективный период выведения 131I и фактор накопления дозы [44]. Ученые стали основоположниками принципа ALARA (As Low As Reasonably Achievable), который был сформулирован в 1954 г. Международной Комиссией по радиологической защите. Данный принцип заключается в минимизации воздействия ионизирующего излучения при достижении эффективности радионуклидной терапии.

## ЗАКЛЮЧЕНИЕ

Прошло более 80 лет с тех пор, как эндокринолог Saul Hertz впервые использовал радиоактивный йод, терапию которым на данный момент получили большое количество пациентов по всему миру.

Современные представления о радиобиологии и дозиметрии в ЯМ признают, что предложенная Hertz методика эффективна и успешно применяется.

Ученые, принимавшие участие в исследованиях в области теоретической физики и ядерного приборостроения, внесли большой вклад в развитие общих представлений о РЙТ.

William Beierwaltes создал базовые принципы использования РЙТ при РЩЖ, которые легли в основу современной молекулярной тераностической концепции. Сегодня основы молекулярной радиотераностики включены в алгоритм принятия клинических решений и выбора тактики ведения пациентов, а молекулярная тераностика становится перспективным направлением. Методы таргетной радионуклидной терапии являются основой для лечения ряда онкологических заболеваний. Будущее персонализированной ЯМ определит интеграция радиотераностики, мультимодальной визуализации, интраоперационной навигации и существующих/новых методов диагностики и лечения в сочетании с прикладными геномными и постгеномными технологиями.

## ДОПОЛНИТЕЛЬНАЯ ИНФОРМАЦИЯ

Источники финансирования.

Госзадание № АААА-А20-120011790174-3 «Радиогеномные предикторы гибридной молекулярной визуализации и радионуклидной терапии эндокринных опухолей».

Конфликт интересов. Авторы декларируют отсутствие явных и потенциальных конфликтов интересов, связанных с публикацией настоящей статьи.

Участие авторов: Все авторы одобрили финальную версию статьи перед публикацией, выразили согласие нести ответственность за все аспекты работы, подразумевающую надлежащее изучение и решение вопросов, связанных с точностью или добросовестностью любой части работы.

Благодарности. Бубнову А.А. — за создание графического изображения.

1. Йод был открыт в 1811 г. французским химиком Bernard Courtois. Ученый извлек йод из пепла морских водорослей. Joseph Gay-Lussac, французский химик и физик, признал, что в ходе эксперимента Courtois получил новый элемент, и назвал его iodes — в переводе с греческого значит фиолетовый. В 1914 г. американскому биохимику Edward Calvin Kendall удалось выделить в кристаллической форме «соединение, содержащее йод, которое присутствует в щитовидной железе». Впоследствии оно было названо тироксином [11]. Позже, в 1917 г., обнаружено, что ЩЖ захватывает йод из крови [12]. Несмотря на эти открытия, физиология ЩЖ оставалась тайной еще несколько десятилетий — до того момента, когда впервые с терапевтической целью были использованы изотопы йода [13].2. В 1930-х годах не существовало комитетов, регулирующих проведение экспериментов на животных. Единственным этическим стандартом являлся личный моральный кодекс исследователей.3. Исследования на кроликах имели решающее значение: они позволили определить количество йода, которое поглощает ЩЖ. Необходимо было установить терапевтическую активность РИ йода, но в ходе эксперимента стало ясно, что 128I и то количество, которое можно произвести с помощью Ra-Be, не подходит для терапевтического применения, так как полученный изотоп имел 25-минутный период полураспада и мог использоваться только для краткосрочных экспериментов [19].4. Во время ранних экспериментов Hertz и Roberts, в 1938–1939 гг., допущен один просчет. Дело в том, что с радиоактивным йодом вводилось определенное количество стабильного йода. Ученые не предполагали, что стабильный йод будет конкурировать с РИ йода за поглощение ЩЖ и тем самым уменьшит проникновение РЙ в ткани. В результате расчеты эффективной дозы для будущих клинических исследований были непомерно высоки и оценены в 27 750 МБк для эффективного лечения тиреотоксикоза [25].5. Уровень метаболизма определяется путем измерения количества кислорода, используемого организмом за определенный промежуток времени. Если измерение производится в состоянии покоя, полученные данные будут показывать уровень базального метаболизма (BMR). Ранее измерение BMR стало одним из первых тестов оценки функции ЩЖ. У пациентов с гипофункцией ЩЖ наблюдался низкий BMR, а у пациентов с гиперфункцией ЩЖ — высокий BMR. Позднее исследования продемонстрировали зависимость BMR от уровня гормонов ЩЖ и показали, что низкий уровень гормонов ЩЖ связан с низким BMR, а высокий уровень — с высоким. Сейчас определение BMR не используют из-за сложности в проведения теста, наличия более достоверных методов исследования, а также из-за того, что BMR подвержен влиянию других факторов кроме функции ЩЖ (например, BMR растет при заболеваниях, сопровождающихся повышением температуры). Нормальный BMR колеблется от -15% до +5%, у пациентов с тиреотоксикозом BMR обычно +20% и более, а при гипотиреозе — BMR -20% и ниже.6. Пациент Keston знал о костных метастазах. Доктора провели сканирование пациента утром 7.12.1941. Определив местонахождение одного метастаза, назначили терапевтическую активность РЙ в размере 370 МКб. Последующее 3-недельное наблюдение показало незначительное поглощение РЙ очагом поражения, демонстрируя положительный эффект от проведенной терапии. Когда в 1944 г. был опубликован отчет о вскрытии пациента, оказалось, что большая часть метастатической опухолевой ткани оказалась недифференцированной, а значит – неспособной накапливать РЙ [31].7. Девушке было всего 20 лет. Хирургами предпринята попытка удалить опухоль ЩЖ. Кроме опухоли ЩЖ наблюдались увеличенные твердые лимфатические узлы с обеих сторон шеи, полная обструкция трахеи и многочисленные вторичные очаги в легких. Удален один лимфатический узел, выполнена трахеостомия. Диагноз подтвержден: карцинома щитовидной железы. После курса РЙТ опухолевая ткань не выявлена. Появилась возможность удалить трахеостомическую трубку. Легкие на рентгенограмме выглядели практически нормальными. Пациентка набрала вес и была здорова уже через 15 месяцев после первого приезда в больницу [34].

## References

[cit1] Al-jubeh W, Shaheen A, Zalloum O. Radioiodine I-131 for diagnosing and treatment of thyroid diseases. Conf. Paper. 2012;6.

[cit2] Jeelani S, Jagat Reddy RC, Maheswaran Thangadurai, Asokan GS, Dany A, Anand B (2014). Theranostics: A treasured tailor for tomorrow. Journal of Pharmacy and Bioallied Sciences.

[cit3] Blahd William H. (2006). Ben cassen and the development of the rectilinear scanner. Seminars in Nuclear Medicine.

[cit4] Collins J. Molecular, Genetic, And Nutritional Aspects Of Major And Trace Minerals. Elsevier Science; 2016.

[cit5] Kaminsky SM, Levy O, Salvador C, et al. The Na+/I- symporter of the thyroid gland. Soc Gen Physiol Ser. 1993;48:251-262. 8503049

[cit6] Dzhikiya E.L., Avilov O.N., Kiseleva Ya.Yu., i dr. Na+/i- simporter (Nis): struktura, funktsii, ekspressiya v norme i opukholyakh // Vestnik RNTsRR. — 2018. — T. 18. — №1. — S. 3.

[cit7] Egorov A.V., Sviridenko N.Yu., Platonova N.M. Osobennosti funktsional'nogo sostoyaniya shchitovidnoi zhelezy posle provedeniya diagnosticheskikh issledovanii s primeneniem iodsoderzhashchikh rentgenokontrastnykh sredstv // Problemy Endokrinologii. — 2005. — T. 51. — №1. — S.50-52. doi: https://doi.org/10.14341/probl200551150-52

[cit8] Wolff J., Chaikoff I.L. (2021). PLASMA INORGANIC IODIDE AS A HOMEOSTATIC REGULATOR OF THYROID FUNCTION. Journal of Biological Chemistry.

[cit9] Akbulut Aylin, Aydinbelge Fadimana Nur, Koca Gökhan (2017). Radioiodine Treatment for Benign Thyroid Diseases. Radionuclide Treatments.

[cit10] Iakovou Ioannis, Giannoula Evanthia, Exadaktylou Paraskevi, Papadopoulos Nikitas (2021). RAI Therapy for Graves’ Hyperthyroidism. Graves' Disease.

[cit11] KENDALL E. C. (2011). THE ISOLATION IN CRYSTALLINE FORM OF THE COMPOUND CONTAINING IODIN, WHICH OCCURS IN THE THYROID. Journal of the American Medical Association.

[cit12] Marine David, Kimball O. P. (2010). THE PREVENTION OF SIMPLE GOITER IN MAN. Nutrition Reviews.

[cit13] van Isselt J.W. Dosage assessment for radioiodine therapy in benign thyroid disorders. Thesis University Utrecht; 2001.

[cit14] Radvanyi Pierre, Villain Jacques (2017). The discovery of radioactivity. Comptes Rendus Physique.

[cit15] FERMI ENRICO (2008). Radioactivity Induced by Neutron Bombardment. Nature.

[cit16] Hertz Barbara E., Schuller Kristin E. (2010). Saul Hertz, MD (1905-1950): A Pioneer in the Use of Radioactive Iodine. Endocrine Practice.

[cit17] Borges de Souza Patricia, McCabe Christopher John (2021). Radioiodine treatment: an historical and future perspective. Endocrine-Related Cancer.

[cit18] Fahey Frederic H., Grant Frederick D., Thrall James H. (2017). Saul Hertz, MD, and the birth of radionuclide therapy. EJNMMI Physics.

[cit19] Ehrhardt Jr John Dennis, Güleç Seza (2020). A Review of the History of Radioactive Iodine Theranostics: The Origin of Nuclear Ontology. Molecular Imaging and Radionuclide Therapy.

[cit20] Compton K. Letter to Saul Hertz: Hertz Family Archive December 15;1936.

[cit21] Hertz S., Roberts A., Evans R. D. (2013). Radioactive Iodine as an Indicator in the Study of Thyroid Physiology.. Experimental Biology and Medicine.

[cit22] JOLIOT F., CURIE I. (2008). Artificial Production of a New Kind of Radio-Element. Nature.

[cit23] Livingood J. J., Seaborg G. T. (2002). Radioactive Iodine Isotopes. Physical Review.

[cit24] Hamilton Joseph G. (2017). THE RATES OF ABSORPTION OF THE RADIOACTIVE ISOTOPES OF SODIUM, POTASSIUM, CHLORINE, BROMINE, AND IODINE IN NORMAL HUMAN SUBJECTS. American Journal of Physiology-Legacy Content.

[cit25] SAWIN CLARK T., BECKER DAVID V. (2009). Radioiodine and the Treatment of Hyperthyroidism: The Early History *. Thyroid.

[cit26] (2008). PROCEEDINGS OF THE THIRTY-FOURTH ANNUAL MEETING OF THE AMERICAN SOCIETY FOR CLINICAL INVESTIGATION HELD IN ATLANTIC CITY, N. J., MAY 4, 1942. Journal of Clinical Investigation.

[cit27] HERTZ SAUL (2011). RADIOACTIVE IODINE IN THE STUDY OF THYROID PHYSIOLOGY. Journal of the American Medical Association.

[cit28] CHAPMAN EARLE M. (2011). THE TREATMENT OF HYPERTHYROIDISM WITH RADIOACTIVE IODINE. Journal of the American Medical Association.

[cit29] Hamilton Joseph G. (2014). The Use of Radioactive Tracers in Biology and Medicine. Radiology.

[cit30] Keston Albert S., Ball Robert P., Frantz V. Kneeland, Palmer Walter W. (2006). Storage of Radioactive Iodine in a Metastasis from Thyroid Carcinoma. Science.

[cit31] FRANTZ V. KNEELAND, BALL ROBERT P., KESTON ALBERT S., PALMER WALTER W. (2006). THYROID CARCINOMA WITH METASTASES. Annals of Surgery.

[cit32] SEIDLIN S. M., OSHRY E., YALOW A. A. (2009). SPONTANEOUS AND EXPERIMENTALLY INDUCED UPTAKE OF RADIOACTIVE IODINE IN METASTASES FROM THYROID CARCINOMA: A PRELIMINARY REPORT*†. The Journal of Clinical Endocrinology & Metabolism.

[cit33] SEIDLIN S. M. (2011). RADIOACTIVE IODINE THERAPY. Journal of the American Medical Association.

[cit34] Smithers D. W. (2008). Some Varied Applications of Radioactive Isotopes to the Localisation and Treatment of Tumours. Acta Radiologica.

[cit35] Hertz S. Use of radioactive iodine in the diagnosis, study and treatment of diseases of the thyroid. Progress in Clinical Endocrinology 1950;65-78

[cit36] DOBYNS BROWN M., VICKERY AUSTIN L., MALOOF FARAHE, CHAPMAN EARLE M. (2009). FUNCTIONAL AND HISTOLOGIC EFFECTS OF THERAPEUTIC DOSES OF RADIOACTIVE IODINE ON THE THYROID OF MAN*. The Journal of Clinical Endocrinology & Metabolism.

[cit37] Pouget Jean-Pierre, Lozza Catherine, Deshayes Emmanuel, Boudousq Vincent, Navarro-Teulon Isabelle (2015). Introduction to Radiobiology of Targeted Radionuclide Therapy. Frontiers in Medicine.

[cit38] Bahn R. Graves’ Disease. New York: Springer; 2015. 344 p.

[cit39] Semenov D.Yu., Boriskova M.E., Farafonova U.V., i dr. Prognosticheskoe znachenie ekspressii natrii-iodnogo simportera dlya vysokodifferentsirovannogo raka shchitovidnoi zhelezy // Klinicheskaya i eksperimental'naya tireoidologiya. — 2015. — T. 11. — №1. — S. 50-58.

[cit40] Spitzweg Christine, Bible Keith C, Hofbauer Lorenz C, Morris John C (2014). Advanced radioiodine-refractory differentiated thyroid cancer: the sodium iodide symporter and other emerging therapeutic targets. The Lancet Diabetes & Endocrinology.

[cit41] Fletcher Alice, Read Martin L., Thornton Caitlin E.M., Larner Dean P., Poole Vikki L., Brookes Katie, Nieto Hannah R., Alshahrani Mohammed, Thompson Rebecca J., Lavery Gareth G., Landa Iñigo, Fagin James A., Campbell Moray J., Boelaert Kristien, Turnell Andrew S., Smith Vicki E., McCabe Christopher J. (2019). Targeting Novel Sodium Iodide Symporter Interactors ADP-Ribosylation Factor 4 and Valosin-Containing Protein Enhances Radioiodine Uptake. Cancer Research.

[cit42] Schlumberger Martin, Brose Marcia, Elisei Rosella, Leboulleux Sophie, Luster Markus, Pitoia Fabian, Pacini Furio (2014). Definition and management of radioactive iodine-refractory differentiated thyroid cancer. The Lancet Diabetes & Endocrinology.

[cit43] MUFAZALOV F. F., SHARIPOVA N. S. (2015). Current status of differetiated radioactive iodine-resistant thyroid cancer: case report of successful long-term treatment with sorafenib. Malignant tumours.

[cit44] Marinelli Leonidas D. (2008). DOSAGE DETERMINATION IN THE USE OF RADIOACTIVE ISOTOPES. Journal of Clinical Investigation.

